# Randomised controlled trial to investigate the effectiveness of the symptom management after radiotherapy (SMaRT) group intervention to ameliorate lower urinary tract symptoms in men treated for prostate cancer

**DOI:** 10.1007/s00520-021-06749-x

**Published:** 2021-12-21

**Authors:** Sara Faithfull, Jane Cockle-Hearne, Agnieszka Lemanska, Sophie Otter, Simon S. Skene

**Affiliations:** 1 School of Health Sciences, Faculty of Health and Medical Sciences, University of Surrey, Guildford, GU2 7XH Surrey UK; 2 Royal Surrey County Hospital, Egerton Road, Guildford, GU2 7XX Surrey UK; 3 Surrey Clinical Trials Unit, University of Surrey, Egerton Road, Guildford, GU2 7XP UK

**Keywords:** Prostate neoplasm, Lower urinary tract symptoms, Symptom management, Rehabilitation radiotherapy, Late effects, Survivorship

## Abstract

**Purpose:**

To evaluate the effectiveness of the symptom management after radiotherapy (SMaRT) group intervention to improve urinary symptoms in men with prostate cancer.

**Methods:**

The randomised controlled trial (RCT) recruited men from one radiotherapy centre in the UK after curative radiotherapy or brachytherapy and with moderate to severe urinary symptoms defined as scores ≥ 8 on the International Prostate Symptom Score (IPSS) questionnaire. Sixty-three men were randomised either; to SMaRT, a 10-week symptom-management intervention including group support, education, pelvic floor muscle exercises, or a care-as-usual group. The primary outcome was the IPSS at 6 months from baseline assessment. Secondary outcomes were IPSS at 3 months, and International Continence Society Male Short Form (ICS), European Organisation for Research and Treatment of Cancer Quality of Life prostate scale (EORTC QLQ-PR25), EORTC QLQ-30 and Self-Efficacy for Symptom Control Inventory (SESCI) at 3 and 6 months from baseline. Analysis of covariance (ANCOVA) was used to analyse the effect of the intervention.

**Results:**

SMaRT group intervention did not improve urinary symptoms as measured by IPSS at 6-months. The adjusted difference was − 2.5 [95%CI − 5.0 to 0.0], *p* = 0.054. Significant differences were detected at 3 months in ICS voiding symptoms (− 1.1 [− 2.0 to − 0.2], *p* = 0.017), ICS urinary incontinence (− 1.0 [− 1.8 to − 0.1], *p* = 0.029) and SESCI managing symptoms domain (13.5 [2.5 to 24.4], *p* = 0.017). No differences were observed at 6 months.

**Conclusions:**

SMaRT group intervention provided short-term benefit in urinary voiding and continence and helped men manage symptoms but was not effective long term.

## Introduction

Prostate cancer (PCa) is one of the most commonly diagnosed cancers in men and accounts for 26% of all new UK male cancer cases [1]. It is estimated that 1.3 million men worldwide are diagnosed per year [2] and with earlier detection and better treatments more men are living with and beyond a PCa diagnosis [3]. Although the quantity of life has improved, the quality of life may be reduced compared to those without cancer because of side effects after treatment [4]. Improving symptom management post-prostate cancer treatment is therefore a priority for research and clinical practice [5].

Quality-of-life in men living with and beyond PCa can be impacted by long-term side effects post-treatment, with the prevalence of erectile dysfunction (87%,) urinary symptoms (20%) and bowel disturbance (14%) occurring up to 12 years after PCa treatment [6]. Two years after initial PCa treatment distress in relation to urinary problems was experienced by 7% of men after radical prostatectomy and 11–16% of men after radiotherapy [6]. In a USA study of Medicare claims, the adjusted risk of grade 2–4 (moderate to severe) urinary symptoms after radiotherapy for PCa was OR 2.49 (95% CI: 2 to 3.11) times that of men without treatment at 10 years [7]. Additionally, a recent UK population study exploring self-reported symptoms and quality of life in 13,097 men 18–42 months post-PCa diagnosis found 13.5% of men reported moderate to severe bother with urinary symptoms and those with urinary bother were more likely to have poorer mental health OR 2.89 (2.54 to 3.27) and severe psychological distress OR 3.69 (3.12–4.38) [8]. Whilst interventions are available for acute symptoms, long-term urinary symptoms after PCa are often poorly addressed reducing men’s ability to socialise and impacting men’s daily activities [9].

Regardless of the type of radiotherapy (external beam radiotherapy or brachytherapy), the close proximity of the genitourinary tract to the prostate means urinary symptoms are relatively common during and shortly after radiotherapy [10]. Acute urinary symptoms are often transient, long-term symptoms can continue for 3–6 months, and late side effects can be newly occurring up to 2 years after external beam radiotherapy [11]. Adverse effects are more severe in those who are older and have poorer physical function and greater urinary symptoms at baseline [12–14]. External beam radiation (EBRT) utilises high-energy photon beams and is shaped and conformed to the profile of the prostate gland such as conformal radiotherapy (CFRT) or delivered through intensity-modulated radiotherapy (IMRT) minimising surrounding normal tissue damage [15]; however, lower doses of radiation can cover a wider field across the pelvis impacting on additional pelvic structures.

Radiation alters bladder contractility through the effect of ionisation on the mucosal-detrusor communication, which impacts on the stability of the bladder and voiding symptoms [16]. Urothelial cells are very radiosensitive and pelvic radiotherapy has both direct as well as bystander affects that result in inflammation, vascular damage and fibrosis [17] causing urinary frequency, bleeding and urinary obstruction [18]. Pelvic floor muscle structures are also affected by radiation with changes in muscle activity and contractility that all impact on urinary function [19]. There is a paucity of studies on conservative intervention approaches for radiation-induced urinary symptoms [10]. Dieperink et al. [20] tested the efficacy of a multidisciplinary rehabilitation intervention, including pelvic floor muscle exercises (PFME), for men during and after external beam radiotherapy. Men in the intervention compared to men in the care-as-usual showed significant improvements in urinary and hormonal symptoms at 20 weeks post-intervention and improved men’s physical quality-of-life. However, one-to-one intervention can be time-consuming, require more clinical resources than group interventions and not provide the opportunity for peer support. Group interventions and self-management support ensure people develop the confidence and skills they need to look after their ongoing physical and mental health [21].

Systematic reviews of the effectiveness of cancer self-management support for cancer survivors have consistently led researchers to call for focused, disease-specific and patient-targeted programmes [22–24]. Previous to the study reported here, our feasibility work found that an augmented symptom management intervention including coaching, bladder retraining and PFME instruction delivered at 3–6 months post-radiotherapy treatment for PCa was feasible within the clinical setting [25]. We hypothesised that in comparison with care-as-usual, at 6 months post-intervention, men who took part in the SMaRT group intervention would report significantly less urinary symptoms, have better symptom-related quality-of-life, less emotional distress and improved confidence to deal with PCa and its associated problems.

## Materials and methods

This study was a two-armed, parallel-group randomised controlled trial. Participants were from one radiotherapy unit, serving four hospitals within NHS England, UK. They had received EBRT with neo-adjuvant or adjuvant androgen deprivation therapy (ADT) or low-dose rate brachytherapy.

### Setting and participants

**Fig. 1:**
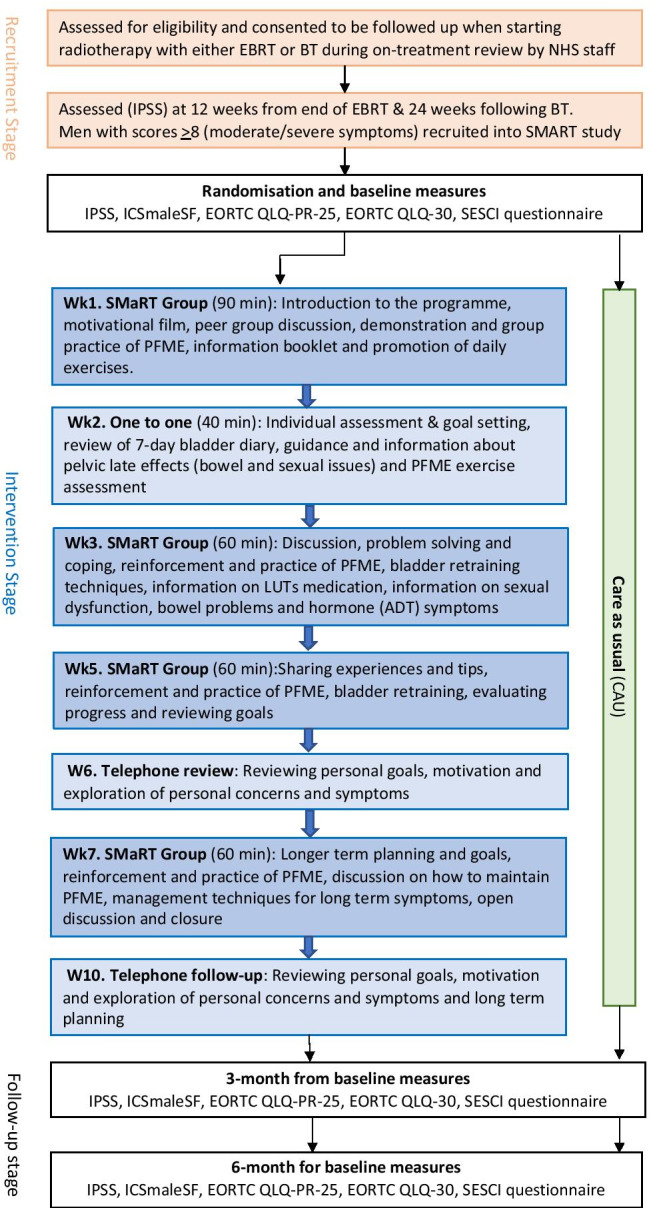
Study flowchart indicating recruitment, intervention components and time points for evaluation measures

Men starting EBRT were asked to participate in the trial during on-treatment physician review. Brachytherapy (BT) patients were invited by letter from their clinical nurse specialist (CNS) after treatment. All participant consent forms were returned by post. Eligibility criteria are summarised in Table 1. Pre-randomisation men completed the International Prostate Symptom Score (IPSS) 12 weeks after EBRT and 24 weeks after BT; this period is considered the recovery period for acute radiotherapy injury and the transition from transient to chronic symptoms. The IPSS formed a screening tool and was used to identify those men who continued to have moderate to severe urinary symptoms (IPSS scores ≥ 8). Men completed baseline study assessments 2 weeks prior to the start of the intervention. Seventy men were subsequently entered into the trial and randomised to receive either the SMaRT group intervention plus care-as-usual or only care-as-usual (Figure 1). Care-as-usual was defined as hospital appointments for surveillance and symptom management with the clinical oncologist and/or telephone support with the CNS*.* Men were stratified for the type of radiotherapy treatment (EBRT vs BT) and randomisation was provided by a registered clinical trials unit. To ensure balance in group sizes, participants were randomly allocated to control or intervention in blocks of 12. Information about treatment, medication, TNM staging and comorbidity was obtained from the medical records.

**Table 1:** SMaRT group intervention study eligibility criteria

Inclusion
* Patients who had:*
• Locally confined prostate cancer disease (up to stage T3BNO)
• and/or received neoadjuvant hormonal therapy.
• Completed external beam radiotherapy 12 weeks prior to the intended commencement of the intervention.
• LDR brachytherapy 24 weeks prior to the intended commencement of the intervention.
• Moderate to severe urinary symptoms defined as a score of ≥ 8 on the International Prostate Symptom Score (IPSS) at 12–24 weeks following EBRT or LDR brachytherapy
• Sufficient understanding of written and spoken English.
Exclusion criteria
* Patients who had:*
• A urinary tract infection.
• A current psychiatric referral.
• A current referral for memory issues/ever been referred to a memory clinic/taking prescribed medication to help with memory.
• Required an interpreter.

### Intervention

The SMaRT group intervention was based on the rehabilitation pathway aimed at reducing the negative impact of treatment-related symptoms and improving function for patients living with and beyond cancer [26]. Specific World Health Organisation rehabilitation recommendations for PCa include pelvic floor muscle re-education, bladder retraining, pharmacotherapy and coping strategies [27]. Adopting effective strategies to cope with prostate cancer provides a foundation for self-management and social support [28]. Peer discussions can also offer emotional support, information exchange and reduce feelings of social isolation [28]. To reflect this, our intervention promoted participant modelling, a key requirement for enhancing self-efficacy [29]. A theory-based, 15-min motivational film was produced by the research team and shown in the first group session to promote group dialogue and peer support [30].

The programme was delivered by an experienced nurse trained in teaching PFME and self-management techniques. PFME were taught both standing, sitting and laying down with 30 min of muscle strength training which included muscle endurance and strength with 10 repetitions for each muscle group. Discussions were conducted on bladder retraining techniques, fluid management, medication and the impact of symptoms on their wellbeing. Modules ran over 10 weeks and comprised four small group sessions (with 5/6 participants), one individual session with the CNS and two telephone sessions with the CNS. This was followed by 4 months of at-home self-management. The group sessions were provided within a community leisure facility; face-to-face individual sessions were conducted at a clinical centre. Information booklets were provided in all the group sessions and set homework was discussed at the following group session. Outcome measures were completed at three time points: 2 weeks prior to the intervention at randomisation (baseline), 3 months and 6 months post-baseline (Figure 1 shows study flowchart indicating recruitment and intervention components).

The *primary outcome* was the sum score of urinary symptoms measured by the IPSS at 6 months post-intervention. *Secondary outcomes* were IPSS at 3 months post-baseline and urinary symptoms measured by the International Continence Society Male Short Form questionnaire (ICSmaleSF); symptom-related quality-of-life measured by the European Organisation for Research and Treatment of Cancer Quality-of-Life scale (EORTC QLQ-PR25), emotional distress measured by the EORTC Quality-of-Life Questionnaire (EORTC QLQ-30); self-efficacy measured by the Self-Efficacy for Symptom Control Inventory (SESCI) at 3 and 6 months post-baseline evaluation.

*IPSS* self-report questionnaire was used as the primary outcome measure as it is a commonly used clinical assessment tool to measure the degree of LUTs and impact on quality of life with seven questions relating to voiding including emptying, frequency, intermittency, urgency, weak stream, straining and nocturia. A score of 7 or less is mildly symptomatic, 8–19 is moderately symptomatic and scores from 20–35 indicate severe symptoms [31].

*ICSmaleSF* a more detailed urinary symptom assessment tool was used to explore urinary functioning and included two distinct LUTs components, voiding (ICSmaleVS) and incontinence (ICSmaleIS). A simple additive score was calculated by adding the 5 items in ICSmaleVC and 6 for ICSmaleIS. Cronbach’s alpha coefficient for this tool was high at 0.76 for voiding and 0.78 for incontinence symptoms against other measures [32]. Both IPSS and ICSmaleSF are generic LUTs measures and not cancer specific; therefore, we included more specific prostate cancer measures.

*EORTC QLQ-PR 25* is designed for use amongst men with localised and metastatic prostate cancer and includes subscale assessing urinary symptoms, bowel symptoms, treatment-related symptoms and sexual functioning, Cronbach’s alpha for urinary and sexual scales 0.70–0.86, for other scales < 0.70 [33].

*EORTC QLQ-C30* for assessing the quality-of-life of cancer patients which is a reliable and valid measure of quality-of-life of cancer patients in multicultural clinical research settings contains five functional scales, global quality-of-life scale and general symptom scales, Cronbach’s alpha across scales 0.52–0.89. This tool is used extensively in clinical research studies worldwide and in our feasibility study [25].

*SESCI* questionnaire measures three dimensions: (i) confidence to perform daily activities; (ii) confidence to cope with urinary symptoms; and (iii) confidence to manage (change) urinary symptoms. Cronbach’s alpha for total scale 0.97 Cronbach’s alpha for each subscale 0.94 [34].

### Sample size calculations and statistical methods

Based on our feasibility study data, a two-sided significance level of 5% and 85% power, a sample size of 21 evaluable participants per arm was considered sufficient to detect a mean difference of change in IPSS score of 4 points between intervention and control, was considered clinically significant. The calculation assumed a standard deviation for change from baseline in IPSS scores of 4.2. To account for possible attrition (withdrawal/loss-to-follow-up) of up to 30%, randomisation was planned to include a minimum of 60 participants.

The primary *statistical analysis* was undertaken using regression methods (analysis of variance, ANCOVA) to estimate the difference in IPSS scores between groups (intervention vs control) at 6 months from randomisation together with a two-sided 95% confidence interval, adjusting for baseline IPSS scores and type of radiotherapy which was included as a covariate. Where 95% confidence intervals (CIs) do not span zero, the results would be regarded as significant.

For secondary outcomes, the differences between the two groups (intervention vs control) were analysed using regression estimates and 95% CIs obtained through the ANCOVA approach outlined above at both the 3 and 6-months follow-up points. The analysis was performed as a complete case analysis. To retain the validity of the randomisation, analyses were undertaken according to the intention-to-treat principle and included all consented and randomised patients for whom outcomes were available.

## Results

### Recruitment and study flow

**Fig. 2:**
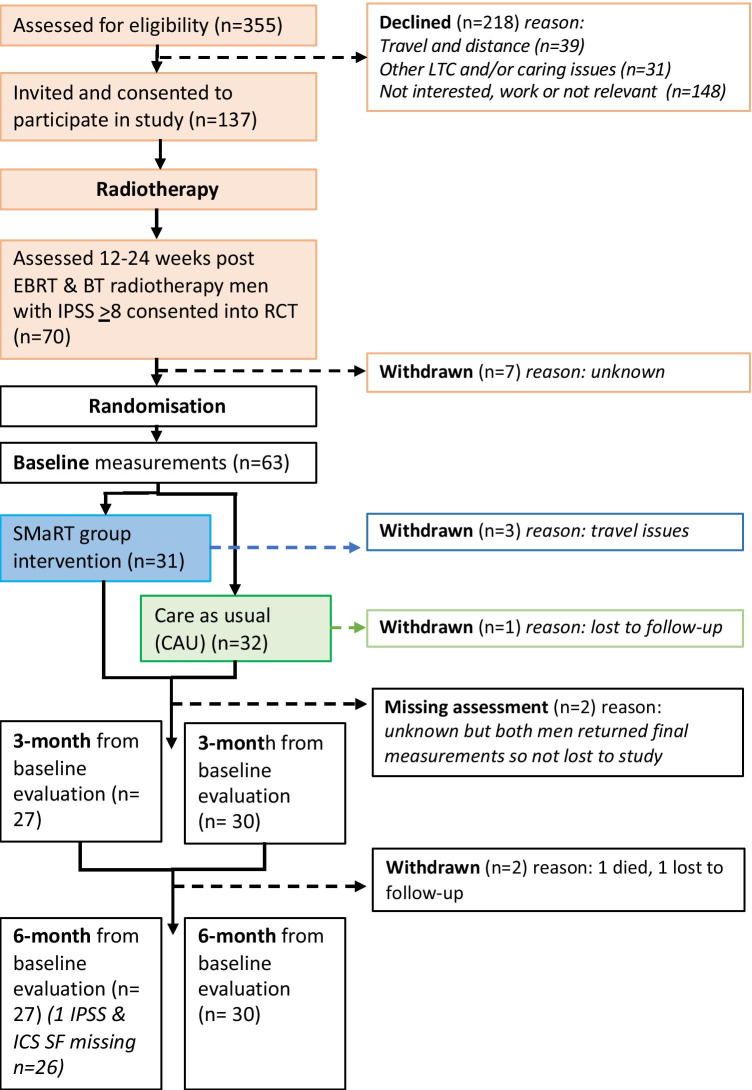
Consort diagram demonstrating recruitment and retention of participants throughout the study

Of 355 invited patients, 137 (39%, 137/355) consented. At screening, 70 consented patients (51%, 70/137) continued to have moderate/severe urinary symptoms (IPSS score ≥ 8) at 12 to 24 weeks post-treatment. Sixty-three men were randomised, 31 to receive the SMaRT group intervention plus usual care and 32 care-as-usual. Three participants in the intervention group withdrew prior to the first session due to travel issues, one control and one intervention participant were lost to follow-up, one intervention participant had missing IPSS scores at final assessment and one control participant died. Figure 2 shows the CONSORT diagram of recruitment and retention of participants through the study. Twenty-eight participants started the intervention; attendance at sessions was 86.2% (mean number of sessions attended 5.45; SD 1.96); study attrition was 9.5% (6/63), excluding follow-up telephone sessions. Overall, telephone follow-up attendance was 63% (attendances: 35/56).

### Baseline characteristics

Demographic, disease and treatment characteristics and screened IPSS scores for the SMaRT and care-as-usual groups at baseline are given in Table 2. The SMaRT group participants were slightly younger than the care-as-usual group; socioeconomic status was high in both groups. The baseline median age score for the sample was 71 (IQR 67 to 76). One or more comorbidities were common with more than 33.3% (21/63) having 2 or more conditions and 25.3% (16/63) having 3 or more conditions. Stage III disease was in 30.1% (19/63) of men and the disease stage was similarly distributed across groups. More men in the care-as-usual group had received ADT 68.7% (22/32) as part of neoadjuvant therapy. Men receiving EBRT made up 77.7% (49/63) of the sample. More men in the SMaRT group received brachytherapy 25.8% (8/31) compared to care-as-usual of 19.4% (6/32). Over 53% (34/63) of the men in the study were taking long-term medication for LUTS. Radiotherapy treatment was adjusted for in the multivariate analysis due to the uneven distribution within the groups.

**Table 2:** Baseline demographic and treatment characteristics, by the randomisation group, of the study population (*n* = 63)

	SMaRT Group *n* = 31	CAU Group *n* = 32	Total (*n* = 63)
Age in years:			
Mean (SD)	69.9 (7.3)	72.2 (6.7)	71.1 (7.1)
Median (IQR)	69 (65.0–74.0)	73 (68.3–77.0)	71 (67–76)
IPSS at baseline:			
Mean (SD)	13.2 (4.0)	13.9 (5.1)	13.6 (4.6)
Median (IQR)	12 (10–17)	12.5 (10.2–16.8)	12 (10.0–16.5)
Social status: EIMD Quintiles: n (%)			
1 Most deprived	0	0	0
2	4 (12.9)	0	4 (1.5)
3	1 (3.2)	5 (15.6)	6 (9.5)
4	4 (12.9)	8 (25.0)	12 (19)
5 Least deprived	22 (71.0)	19 (59.4)	41 (65)
Missing	0	1 (3.1)	1 (1.5)
Body mass index (BMI) Kgm^2^: *n* (%)			
< 18.5	1 (3.2)	0	1 (1.5)
18.5–24.9	3 (9.6)	1 (3.1)	4 (6.3)
25–29.9	5 (16.1)	7 (21.8)	12 (19)
> 30	1 (3.2)	3 (9.3)	4 (6.3)
Missing	21(67.7)	21 (65.6)	42 (66.6)
Comorbidities: *n* (%)			
None	1 (3.2)	3 (9.3)	4 (6.3)
1	5 (16.1)	4 (12.5)	9 (14.2)
2	9 (29.0)	12 (37.5)	21 (33.3)
3	10 (32.3)	6 (18.7)	16 (25.3)
4	4 (12.9)	1 (3.1)	5 (7.9)
5	2 (6.5)	4 (12.5)	6 (9.5)
6	0	1 (3.1)	1 (1.5)
8–9	3 (9.7)	3 (9.3)	3 (4.7)
Missing	0	1 (3.1)	1 (1.5)
Stage of disease: *n* (%)			
I	9 (29.0)	8 (25)	17 (26.9)
II	7 (22.5)	11 (34.3)	18 (28.5)
III	10 (32.2)	9 (28.5)	19 (30.1)
Missing	5 (16.1)	4 (12.5)	9 (14.2)
Prostate cancer therapy			
Androgen deprivation therapy: n (%)	14 (45.1)	22 (68.7)	36 (57.1)
Radiotherapy			
EBRT	23 (74.1)	26 (81.3)	49 (77.7)
LDR brachytherapy	8 (25.8)	6 (19.4)	14 (22.2)
EBRT Dose: *n* (%)			
55 Gy	0	1 (3.1)	1 (1.5)
74 Gy	19 (61.2)	23 (71.8)	42 (66.6)
≥ 76 Gy	1 (3.2)	2 (6.2)	2 (3.1)
Missing dose data	11 (35.4)	6 (18.7)	17 (26.9)
EBRT Fraction: n(%)			
20	0	1 (3.1)	1 (1.5)
35	1(3.2)	0	1 (1.5)
37	19(61.2)	25 (78.1)	44 (69.8)
Missing	11(35.4)	6 (18.75)	17 (26.9)
Time since EBRT (months): *n*%			
3–4	2 (6.4)	6 (18.7)	8 (1.5)
5–6	13 (41.9)	10 (31.2)	23 (36.5)
7–8	4 (12.9)	5 (15.6)	9 (14.2)
9–10	4 (12.9)	5 (15.6)	9 (14.2)
Time since LDR brachytherapy (months): *n*%			
4–5	3 (9.6)	0	3 (4.7)
6–7	2 (6.4)	4 (12.5)	6 (9.5)
8–10	3 (9.6)	2 (6.2)	5 (7.9)
Taking medication for LUTs: *n* (%)	16 (51.6)	19 (59.3)	34 (53.9)
Alpha blocker (Tamsulosin)	15 (48.3)	18 (56.2)	33 (52.3)
Anti-muscarinic (Solifenacin)	1 (3.2)	1 (3.1)	2 (3.1)

*SD*, standard deviation; *Gy*, Gray; *QR*, inter-quartile range; *EIMD*, English Index of Multiple Deprivation; *EBRT*, external beam radiation therapy; *LDR*, low-dose rate; *LUTS*, lower urinary tract symptoms; *IPSS*, International Prostate Symptom Scale

**Fig. 3:**
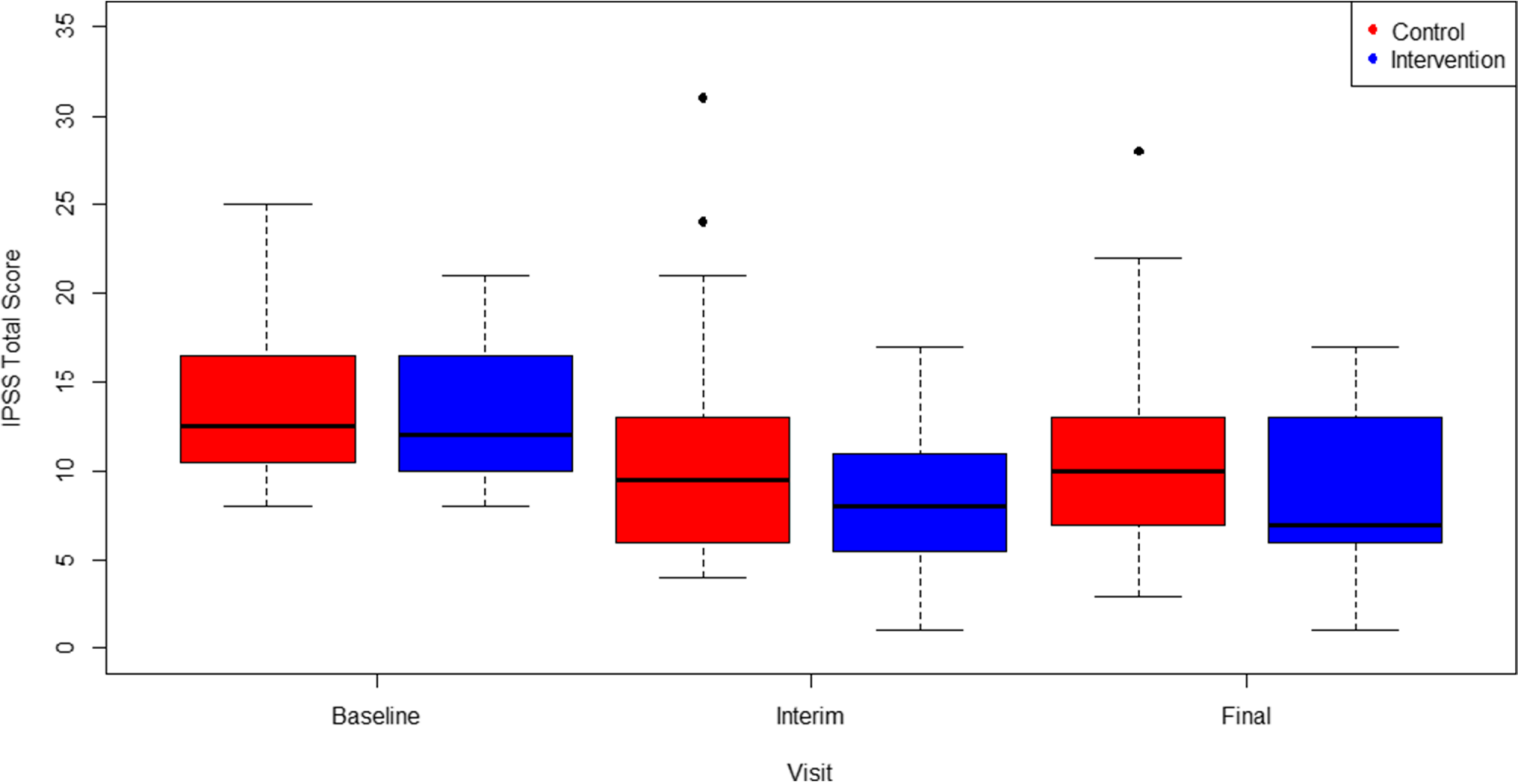
Distribution of IPSS Total Scores at each time point (interim 3 months from baseline & final 6 months from baseline) for the SMaRT group intervention compared to CAU (control). Abbreviations: *SMaRT*, Symptom Management after Radiotherapy group intervention; *CAU*, Care As Usual control; *IPSS*, International Prostate Symptom Score

Box plots (Figure 3) illustrate a decrease in IPSS scores for both groups from baseline to 3 and 6 months; there was a trend for reduction in IPSS with the SMaRT group at both time points, but it was not significant which may be partly due to the small sample size. However, there was a large overlap in observed values between the groups. We found no significant differences in our primary outcome between the SMaRT group and care-as-usual groups in scores on the IPSS at 3 or 6 months even when adjusted for pre-intervention IPSS baseline scores and adjustment of radiotherapy type (Table 3).

**Table 3:** Primary and secondary outcome scores between baseline and 3 and 6 months with adjusted change scores

	Baseline	3 months from baseline	Change	Adjusted difference*	*p*-value	6 months from baseline	Change	Adjusted difference*	*p*-value
**IPSS**									
CAU	13.9 (5.1)	10.8 (6.1)	− 3.1 (− 4.9 to − 1.3)			11.0 (6.1)	− 2.6 (− 4.6 to − 0.6)		
SMaRT	13.2 (4.0)	8.6 (4.3)	− 5.0 (− 6.5 to − 3.4)	− 2.1 (− 4.2 to 0.1)	0.066	8.7 (4.7)	− 4.8 (− 6.7 to − 3.0)	− 2.5 (− 5.0 to 0.0)	0.054
**ICS voiding symptoms**									
CAU	5.6 (2.7)	4.9 (3.1)	− 0.8 (− 1.3 to − 0.2)			4.6 (2.4)	− 0.9 (− 1.5 to − 0.3)		
SMaRT	5.5 (2.7)	3.9 (2.0)	− 1.9 (− 2.6 to − 1.1)	− 1.1 (− 2.0 to − 0.2)	**0.017**	4.3 (2.8)	− 1.3 (− 2.2 to − 0.3)	− 0.3 (− 1.3 to 0.7)	0.521
**ICS incontinence symptoms**									
CAU	2.8 (1.9)	3.2 (2.1)	0.5 (− 0.2 to 1.2)			3.1 (2.5)	0.3 (− 0.4 to 1.0)		
SMaRT	3.4 (2.2)	2.7 (1.5)	− 0.9 (− 1.6 to − 0.2)	− 1.0 (− 1.8 to − 0.1)	**0.029**	2.7 (1.6)	− 0.9 (− 1.9 to − 0.1)	− 0.9 (− 1.9 to 0.1)	0.073
**EORTC25 urinary domain**									
CAU	27.7 (15.6)	22.3 (17.9)	− 4.9 (− 9.3 to − 0.6)			21.3 (16.3)	− 6.6 (− 11.6 to − 1.6)		
SMaRT	27.3 (15.9)	20.8 (14.6)	− 7.1 (− 11.2 to − 3.0)	− 1.9 (− 7.5 to 3.8)	0.506	18.5 (15.0)	− 9.7 (− 14.9 to − 4.5)	− 3.7 (− 10.0 to 2.6)	0.245
**EORTC30 emotional functioning domain**									
CAU	84.7 (14.4)	87.7 (15.2)	2.9 (− 1.8 to 7.5)			87.6 (14.9)	2.4 (− 1.4 to 6.2)		
SMaRT	85.7 (17.0)	83.3 (18.2)	− 2.5 (− 7.8 to 2.7)	− 5.0 (− 11.7 to 1.8)	0.147	87.7 (13.6)	1.4 (− 1.9 to 4.8)	− 0.3 (− 5.1 to 4.5)	0.902
**SESCI performing daily activities**									
CAU	89.7 (16.4)	83.3 (25.3)	− 3.9 (− 10.7 to 2.9)			87.4 (20.9)	− 1.2 (− 6.6 to 4.2)		
SMaRT	88.3 (17.9)	85.7 (20.5)	− 3.3 (− 10.8 to 4.1)	0.6 (− 9.1 to 10.3)	0.901	86.1 (21.6)	− 4.5 (− 8.9 to − 0.2)	− 3.5 (− 10.4 to 3.5)	0.324
**SESCI coping with symptoms**									
CAU	77.8 (19.0)	78.0 (20.8)	− 4.8 (− 13.5 to 3.9)			80.9 (17.3)	1.9 (− 4.0 to 7.7)		
SMaRT	74.3 (18.6)	77.5 (17.5)	2.8 (− 1.8 to 7.3)	5.4 (− 4.5 to 15.0)	0.274	83.2 (15.5)	7.0 (3.4 to 10.7)	4.3 (− 2.2 to 10.8)	0.192
**SESCI managing symptoms**									
CAU	67.1 (21.1)	63.0 (24.9)	− 5.9 (− 14.7 to 2.9)			66.8 (22.9)	− 0.1 (− 5.6 to 5.5)		
SMaRT	58.6 (22.5)	72.5 (20.6)	11.0 (3.2 to 18.9)	13.5 (2.5 to 24.4)	**0.017**	71.0 (22.6)	8.8 (0.9 to 16.6)	7.0 (− 2.2 to 16.0)	0.133

^*^Adjusting for baseline IPSS scores and Radiotherapy (EBRT vs BT) which were included as covariates

Abbreviations: *CAU*, care as usual; *SMaRT*, Symptom-Management After Radiotherapy group intervention; *IPSS*, International Prostate Symptom Scale; *ICS*, International Continence Scale, EORTC European; SECSI; *p*-values that are significant at the 0.05 level

At 3 months, ICS voiding symptoms had improved by − 1.9 points (95% CI: − 2.6 to − 1.1) in the SMaRT group and by − 0.8 points (95% CI − 1.3 to − 0.2) in the care-as-usual group, a significant adjusted difference of − 1.1 points (− 2.0 to − 0.2) favouring SMaRT group (*p* = 0.017 Table 3). ICS voiding symptoms did not differ significantly at 6 months. At 3 months, ICS incontinence symptoms had improved by − 0.9 points (− 1.6 to − 0.2) in the SMaRT group and deteriorated by 0.5 points (− 0.2 to 1.2) in the care-as-usual group, a significant adjusted difference of − 1.0 points (− 1.8 to − 0.1) favouring SMaRT group (*p* = 0.029) (Table 3). ICS incontinence symptoms did not differ significantly at 6 months. There were no observed differences in quality-of-life (EORTC QLQ-C30) or urinary domain scores (EORTC QLQ-PR25) between groups.

Self-efficacy for managing symptoms measured by the SESCI improved by 11.0 points (95% CI: 3.2 to 18.9) in the SMaRT group and decreased by − 5.9 points (− 14.7 to 2.9) in the care-as-usual group, a significant adjusted difference of 13.9 points (2.5 to 24.4) favouring the SMaRT group (*p* = 0.017). Self-efficacy for managing symptoms did not differ significantly between the groups at 6 months. We noted no significant differences between the care-as-usual and SMaRT groups in self-efficacy for performing daily activities or self-efficacy for coping with symptoms at either 3 months or 6 months.

## Discussion

We found that symptom management after the radiotherapy (SMaRT) group intervention had no significant difference on IPSS outcomes in men who had received radiotherapy for PCa, compared to care-as-usual, at 3 and 6 months, but did provide significant differences in domain-specific urinary symptoms on ICS voiding and incontinence at 3 months. Our intervention improved urinary symptoms in ICS voiding by − 1.9 and when adjusted for baseline scores a change of − 1.1 and urinary incontinence by − 0.9. As secondary outcomes, we observed significant benefit in reported self-efficacy for men in managing symptoms at 3 months with a 13.5 (2.5 to 24.4) adjusted point difference in the SMaRT group compared to − 5.9 (− 14.7 to 2.9) care-as-usual. The intervention effect was not able to be sustained beyond 3 months as seen in the follow-up scores where there was little difference between groups.

Contrary to our findings, Dieperink et al. [20] in their study of multidisciplinary rehabilitation found a 5.8 point (Cohen’s *d* = 0.40; p0.011) difference in urinary sum scores for irritative symptoms between those receiving the intervention and care-as-usual recorded at 6 months post-radiotherapy. In this study, the usual care group had 1 physician visit face to face 4 weeks after radiotherapy, whereas our care-as-usual group saw the physician at 6 months and had ongoing contact with a CNS. Despite this difference, the change in urinary scores was not at the same level as that found by Dieperink [20] or in our feasibility study [25]. The distinct difference between these studies is the intervention intensity; SMaRT was primarily group based and may not have provided the individualised approach provided by Dieperink [20] in the face-to-face multidisciplinary rehabilitation setting. This dosing effect is important in PFME as variation in delivery such as the muscle-targeted intensity of the programme and the position in which pelvic floor muscle contraction is taught, are influential factors and contribute to variation in intervention outcomes [35]. Previous studies provided interventions for PCa from the start of radiotherapy as a way of preventing adverse effects and managing any pre-treatment symptoms [20, 36]. Providing SMaRT group intervention over a longer time period may prove more effective, than waiting until after radiotherapy is completed, tackling LUTs before symptoms are chronic, pelvic floor musculature compromised and coping strategies established. Furthermore, focusing on those men at higher risk of long-term urinary consequences pre-treatment and providing prehabilitation may also enhance the intervention.

There is a need to focus more on the mechanistic science underpinning interventions for managing pelvic radiotherapy late effects. Damage to pelvic floor vasculature and fibrosis all contribute to lower urinary tract symptoms [18]. One retrospective study of men with PCa who underwent MRI before and after EBRT or BT showed significant reductions in urethral length, increased signal intensity of the obturator internus muscle and peri-urethral part of the levator ani, suggestive of fibrotic changes [16]. Diepernick et al.(37) in a follow-on study found that that pelvic floor muscle strength of men in their intervention study diminished over the 3 years post-intervention but that men still had better LUTS than men in their control group.

The evidence for lifestyle interventions for reducing PCa treatment adverse effects is growing [38, 39]. Furthermore, whilst a recent systematic review [40] has classified the important components of benign LUTS self-management, the active components or behavioural interventions that contribute to these are far from clear. Skolarus et al. [36] reported a RCT of a self-management programme for prostate cancer survivors and found no significant differences between intervention and control groups. However, like our study, coping appraisal was higher (2.8 vs 2.6 *p* = 0.02) in men who had received the intervention. This highlights the problems with a heterogeneity of the needs of men, specificity of intervention and how best to measure the clinical significance of any benefits of self-management, i.e. is it the symptoms that are the primary aim or the self-efficacy? In our study, men had a high level of self-efficacy across domains from the start of the study but clearly the participant modelling and information helped them manage symptoms and feel more confident post-treatment.

### Strengths and limitations

A strength of the SMaRT study was we were able to map men’s urinary symptoms over time, prior to randomisation, allowing us to target those in greatest need of intervention and control for baseline scores. We focused on those with moderate to severe symptoms but clearly the intervention maybe helpful also for men with less severe urinary symptoms. Focusing on those with chronic symptoms may reflect a more difficult population that as urinary symptoms continue after radiotherapy, they can become more intractable [18]. Men who have brachytherapy are much more likely to have issues with voiding due to swelling and inflammation which are probably less affected by PFME; however, this was adjusted for in the analysis. A limitation of our study is that we did not use, surface anal electromyography (EMG) to assess men’s pelvic floor, or provide ongoing data on pelvic floor changes, or participant diaries to record adherence to PFME. Given EMG assessment is invasive in a group setting, it may have been useful to use it in the one-to-one session with the CNS to assess the effectiveness of the individuals’ exercises. Studies of PFME in men with PCa have focused mainly on the surgical setting and have shown that pelvic floor muscle exercises pre- and post-treatment can improve symptom outcomes [41] and this evidence is reflected in NICE UK [42] prostate cancer guidelines. Studies show that men who continue to have LUTS after radiotherapy have significant reductions in quality-of-life [43]. In a systematic review of 13 studies, post-radiotherapy pelvic floor muscle changes were found to occur between 2 and 26 months after radiation, showing the wide range of individual response in men with PCa [19]. Some of this variance may be due to prior LUTS [14]; however, we adjusted for this as part of our analysis.

## Conclusions

The study showed that the SMaRT group intervention helped men feel more confident in managing symptoms and created small changes in LUTS but was not clinically significant or sustained. Evidence for conservative interventions that augment symptom management post-pelvic radiotherapy is limited; therefore, this RCT provides important evidence that contributes to improving treatment pathways for those living with and beyond prostate cancer. The growing number of men now surviving and requiring long-term symptom management for consequences of PCa has contributed to the growth in supported self-management programs to address long-term survivorship care [44–46] but the outcomes of these studies are varied partly because symptom management requires targeted interventions to improve not only self-efficacy but personalised management strategies to improve outcomes. Although SMaRT group intervention was not effective long-term, some of the elements show promise and that a more targeted one-to-one and earlier intervention approach may be needed to address the more complex LUTS as a result of radiotherapy.

## Data Availability

The data can be requested by contact the corresponding author. The access will be granted subject to a reasonable request and data sharing agreement.

## Code availability

No bespoke software was created.

## References

[1] Cancer Research UK Prostate Cancer survival statistics London 2015-17. Available from: https://www.cancerresearchuk.org/health-professional/cancer-statistics/statistics-by-cancer-type/prostate-cancer. Accessed 11 Aug 2021

[2] Bray F, Ferlay J, Soerjomataram I, Siegel RL, Torre LA, Jemal A (2018). Global cancer statistics 2018: GLOBOCAN estimates of incidence and mortality worldwide for 36 cancers in 185 countries. CA Cancer J Clin..

[3] Hoffman KE, Penson DF, Zhao Z, Huang L-C, Conwill R, Laviana AA (2020). Patient-reported outcomes through 5 years for active surveillance, surgery, brachytherapy, or external beam radiation with or without androgen deprivation therapy for localized prostate cancer. JAMA..

[4] Mazariego CG, Egger S, King MT, Juraskova I, Woo H, Berry M (2020). Fifteen year quality of life outcomes in men with localised prostate cancer: population based Australian prospective study. BMJ..

[5] Lagergren P, Schandl A, Aaronson NK, Adami HO, de Lorenzo F, Denis L (2019). Cancer survivorship: an integral part of Europe’s research agenda. Mol Oncol..

[6] Carlsson S, Drevin L, Loeb S, Widmark A, Lissbrant IF, Robinson D (2016). Population-based study of long-term functional outcomes after prostate cancer treatment. BJU Int..

[7] Kim S, Moore DF, Shih W, Lin Y, Li H, Shao YH (2013). Severe genitourinary toxicity following radiation therapy for prostate cancer–how long does it last?. J Urol..

[8] Wilding S, Downing A, Wright P, Selby P, Watson E, Wagland R (2019). Cancer-related symptoms, mental well-being, and psychological distress in men diagnosed with prostate cancer treated with androgen deprivation therapy. Qual Life Res..

[9] Paterson C, Jones M, Rattray J, Lauder W (2013). Exploring the relationship between coping, social support and health-related quality of life for prostate cancer survivors: a review of the literature. Eur J Oncol Nurs..

[10] Liberman D, Mehus B, Elliott SP (2014). Urinary adverse effects of pelvic radiotherapy. Transl Androl Urol..

[11] Faithfull S, Lemanska A, Aslet P, Bhatt N, Coe J, Drudge-Coates L (2015). Integrative review on the non-invasive management of lower urinary tract symptoms in men following treatments for pelvic malignancies. Int J Clin Pract.

[12] Lemanska A, Dearnaley DP, Jena R, Sydes MR, Faithfull S (2018). Older age, early symptoms and physical function are associated with the severity of late symptom clusters for men undergoing radiotherapy for prostate cancer. Clin Oncol (R Coll Radiol).

[13] Chen RC, Clark JA, Talcott JA (2009). Individualizing quality-of-life outcomes reporting: how localized prostate cancer treatments affect patients with different levels of baseline urinary, bowel, and sexual function. J Clin Oncol.

[14] Groom N, Tsang Y, Lowe G, Hoskin P (2021). Risk factors for urethral stricture following external beam radiotherapy and HDR brachytherapy for prostate cancer. Brachytherapy.

[15] Tree A, Khoo V (2009). Treatment of early prostate cancer: radiotherapy, including brachytherapy. Trends Urol Gynaecol Sex Health..

[16] Marigliano C, Donati OF, Vargas HA, Akin O, Goldman DA, Eastham JA (2013). MRI findings of radiation-induced changes in the urethra and periurethral tissues after treatment for prostate cancer. Eur J Radiol..

[17] Awad MA, Gaither TW, Osterberg EC, Murphy GP, Baradaran N, Breyer BN (2018). Prostate cancer radiation and urethral strictures: a systematic review and meta-analysis. Prostate Cancer Prostatic Dis..

[18] Bosch R, McCloskey K, Bahl A, Arlandis S, Ockrim J, Weiss J (2020). Can radiation-induced lower urinary tract disease be ameliorated in patients treated for pelvic organ cancer: ICI-RS 2019?. Neurourol Urodyn..

[19] Bernard S, Ouellet MP, Moffet H, Roy JS, Dumoulin C (2016). Effects of radiation therapy on the structure and function of the pelvic floor muscles of patients with cancer in the pelvic area: a systematic review. J Cancer Surviv..

[20] Dieperink K, Johansen C, Hansen S, Wagner L, Anderson K, Minet L (2013). The effects of multidisciplinary rehabilitation: RePCa- a randomnised study among primary prostate cancer patients. Br J Cancer..

[21] de Longh A, Fagan P, Fenner J, Kidd L (2015) A practical guide to self-management support. 90 Long Acre, London, WC2E 9RA: The Health Foundation. Contract No.: ISBN 978-1-906481-74-4

[22] Cuthbert CA, Farragher JF, Hemmelgarn BR, Ding Q, McKinnon GP, Cheung WY (2019). Self-management interventions for cancer survivors: a systematic review and evaluation of intervention content and theories. Psycho-Oncol..

[23] Boland L, Bennett K, Connolly D (2018). Self-management interventions for cancer survivors: a systematic review. Support Care Cancer..

[24] Kim SH, Kim K, Mayer DK (2017). Self-management intervention for adult cancer survivors after treatment: a systematic review and meta-analysis. Oncol Nurs Forum..

[25] Faithfull S, Cockle-Hearne J, Khoo V (2011). Self-management after prostate cancer treatment: evaluating the feasibility of providing a cognitive and behavioural programme for lower urinary tract symptoms. BJU Int..

[26] Macmillan Cancer Support (2018). Cancer rehabilitation pathways.

[27] Stout NL, Santa Mina D, Lyons KD, Robb K, Silver JK (2021). A systematic review of rehabilitation and exercise recommendations in oncology guidelines. CA Cancer J Clin..

[28] Paterson C, Roberts C, Toohey K, McKie A (2020). Prostate cancer prehabilitation and the importance of multimodal interventions for person-centred care and recovery. Semin Oncol Nurs..

[29] Foster C, Breckons M, Cotterell P, Barbosa D, Calman L, Corner J (2015). Cancer survivors’ self-efficacy to self-manage in the year following primary treatment. J Cancer Surviv..

[30] Cockle-Hearne J, Cooke D, Faithfull S (2016). Developing peer support in film for cancer self-management: what do men want other men to know?. Support Care Cancer..

[31] Barry MJ, Fowler Jnr FJ, O’Leary MP, Bruskewitch RC, Holtgrewe HL, Mebust WK (1992). The American Urological Association Symptom Index for Benign Prostatic Hyperplasia: The Measurement Committee of the American Urological Association. J Urol..

[32] Donovan JL, Peters TJ, Abrams P, Brookess ST, de la Rosette JJMCH, Schafer W (2000). Scoring the Short Form ICSmaleSF Questionnaire: International Continence Society. J Urol.

[33] Whistance RN, Conroy T, Chie W, Costantini A, Sezer O, Koller M, et al. (2009) Clinical and psychometric validation of the EORTC QLQ-CR29 questionnaire module to assess health-related quality of life in patients with colorectal cancer. European Journal Of Cancer (Oxford, England: 1990) 45(17):3017–26 10.1016/j.ejca.2009.08.014 19765978

[34] Campbell LC, Keefe FJ, McKee DC, Edwards CL, Herman SH, Johnson LE (2004). Prostate cancer in African Americans: relationship of patient and partner self-efficacy to quality of life. J Pain Symptom Manage..

[35] Hall LM, Aljuraifani R, Hodges PW (2018). Design of programs to train pelvic floor muscles in men with urinary dysfunction: systematic review. Neurourol Urodyn..

[36] Skolarus TA, Metreger T, Wittmann D, Hwang S, Kim HM, Grubb RL (2019). Self-management in long-term prostate cancer survivors: a randomized, controlled trial. J Clin Oncol..

[37] Dieperink KB, Hansen S, Wagner L, Minet LR, Hansen O (2021). Long-term follow-up 3 years after a randomized rehabilitation study among radiated prostate cancer survivors. J Cancer Surviv.

[38] Zuniga KB, Chan JM, Ryan CJ, Kenfield SA (2020). Diet and lifestyle considerations for patients with prostate cancer. Urol Oncol..

[39] Geerkens MJM, Pouwels NSA, Beerlage HP (2020). The effectiveness of lifestyle interventions to reduce side effects of androgen deprivation therapy for men with prostate cancer: a systematic review. Qual Life Res..

[40] Albarqouni L, Sanders S, Clark J, Tikkinen KAO, Glasziou P (2021). Self-management for men with lower urinary tract symptoms: a systematic review and meta-analysis. Ann Fam Med..

[41] Zhang A, Fu A, Moore S, Zhu H, Strauss G, Kresevic D (2017). Is a behavioral treatment for urinary incontinence beneficial to prostate cancer survivors as a follow-up care?. J Cancer Survivorship.

[42] NICE (2019) Prostate cancer: diagnosis and management. London: National Institute for Clinical Evidence. Contract No.: ng131

[43] Downing A, Wright P, Hounsome L, Selby P, Wilding S, Watson E (2019). Quality of life in men living with advanced and localised prostate cancer in the UK: a population-based study. Lancet Oncol..

[44] Frankland J, Brodie H, Cooke D, Foster C, Foster R, Gage H (2019). Follow-up care after treatment for prostate cancer: evaluation of a supported self-management and remote surveillance programme. BMC Cancer..

[45] Skolarus TA, Wittmann D, Hawley ST (2017). Enhancing prostate cancer survivorship care through self-management. Urol Oncol..

[46] Bowler M, Dehek R, Thomas E, Ngo K, Grose L (2019). Evaluating the impact of post-treatment self-management guidelines for prostate cancer survivors. J Med Imaging Radiat Sci..

